# Relationship Between Aquatic Fungal Diversity in Surface Water and Environmental Factors in Yunnan Dashanbao Black-Necked Crane National Nature Reserve, China

**DOI:** 10.3390/jof11070526

**Published:** 2025-07-16

**Authors:** Kaize Shen, Yufeng Tang, Jiaoxu Shi, Zhongxiang Hu, Meng He, Jinzhen Li, Yuanjian Wang, Mingcui Shao, Honggao Liu

**Affiliations:** 1 School of Agronomy and Life Sciences, Zhaotong University, Zhaotong 657000, China; ztushenkz@163.com (K.S.); 34013@ztu.edu.cn (J.S.); 47038@ztu.edu.cn (Z.H.); hm3127546274@163.com (M.H.); shari_1001@163.com (J.L.); 2 Yunnan Key Laboratory of Gastrodia and Fungi Symbiotic Biology, Zhaotong University, Zhaotong 657000, China; 3 Yunnan Engineering Research Center of Green Planting and Processing of Gastrodia, Zhaotong University, Zhaotong 657000, China; 4 School of Water Resources and Environment, China University of Geosciences (Beijing), Beijing 100083, China; 5 Management Bureau of Yunnan Dashanbao Black-Necked Crane National Nature Reserve, Zhaotong 657000, China; wyj77880484@163.com (Y.W.); smc593468245@163.com (M.S.)

**Keywords:** Yunnan Dashanbao Black-necked Crane National Nature Reserve, aquatic fungi, environmental factors, fungal diversity, community composition, trophic guilds

## Abstract

Aquatic fungi serve as core ecological engines in freshwater ecosystems, driving organic matter decomposition and energy flow to sustain environmental balance. Wetlands, with their distinct hydrological dynamics and nutrient-rich matrices, serve as critical habitats for these microorganisms. As an internationally designated Ramsar Site, Yunnan Dashanbao Black-Necked Crane National Nature Reserve in China not only sustains endangered black-necked cranes but also harbors a cryptic reservoir of aquatic fungi within its peat marshes and alpine lakes. This study employed high-throughput sequencing to characterize fungal diversity and community structure across 12 understudied wetland sites in the reserve, while analyzing key environmental parameters (dissolved oxygen, pH, total nitrogen, and total phosphorus). A total of 5829 fungal operational taxonomic units (OTUs) spanning 649 genera and 15 phyla were identified, with *Tausonia* (4.17%) and *Cladosporium* (1.89%) as dominant genera. Environmental correlations revealed 19 genera significantly linked to abiotic factors. FUNGuild functional profiling highlighted saprotrophs (organic decomposers) and pathogens as predominant trophic guilds. Saprotrophs exhibited strong associations with pH, total nitrogen, and phosphorus, whereas pathogens correlated primarily with pH. These findings unveil the hidden diversity and ecological roles of aquatic fungi in alpine wetlands, emphasizing their sensitivity to environmental gradients. By establishing baseline data on fungal community dynamics, this work advances the understanding of wetland microbial ecology and informs conservation strategies for Ramsar sites.

## 1. Introduction

Fungi are incredibly diverse organisms found in nearly every habitat on Earth, with estimates suggesting there are between 2.2 and 3.8 million species in total [1]. Freshwater fungi constitute a highly diverse ecological group within this kingdom. Fungi serve as integrative regulators of aquatic ecosystem processes, orchestrating four interconnected functional domains: (i) Biogeochemical Cycling—mineralizing refractory organic polymers (e.g., lignin) into bioavailable nutrients through extracellular enzymatic hydrolysis [2]; (ii) Energy Pathway Regulation—channeling energy from inedible biomass to higher trophic levels via parasitic mycoloops (e.g., Chytridiomycota-zooplankton linkages) [3]; (iii) Habitat Engineering—stabilizing sediments through hyphal networks and creating micro-oxygen zones for anaerobic microbiota [4]; (iv) Stress Response Mediation—suppressing greenhouse gas emissions via substrate competition (e.g., trehalose degradation) and degrading xenobiotics through non-specific oxidation [5]. Shearer [6] reported 288 ascomycete species in watery settings, a number that has expanded to 3077 species [7]. However, many more species are yet to be discovered. Aquatic fungi are broadly defined as relying on an aquatic habitat for all or part of their life cycle [8]. Aquatic fungi have important functions in freshwater ecosystems by contributing to material cycling and energy flow [9,10], making them a focus for the discovery and development of biologically active substances [11]. Aquatic fungi include Ascomycota, Basidiomycota, Chytridiomycota, Blastocladiomycota, Hyphochytriomycota, Labyrinthulomycota, and Zygomycota [12]. Approximately 3000 different fungal species are associated with aquatic habitats worldwide [1,8]. However, because of methodological limitations and a small scientific community, systematic research of fungal diversity and its ecological significance in aquatic ecosystems has encountered difficulties. Most research on fungal diversity has focused on genetic and molecular studies from soil environments [13,14]. However, the variety, quantitative abundance, ecological function, and interactions of aquatic fungi with environmental elements [15] are mostly unknown, speculative, and absent from general theories of aquatic ecology and biogeochemistry [3,16,17]. Environmental variables can affect fungal community composition and function [18,19]. Therefore, there is an urgent need to assess fungal diversity in freshwater and its relationship to environmental factors [20,21].

Yunnan Dashanbao Black-Necked Crane National Nature Reserve (hereafter “Dashanbao Nature Reserve”), situated in Zhaotong City, Northwest Yunnan Province of China (27°8′38″–27°28′42″ N, 103°14′55″–103°18′38″ E), encompasses a high-altitude wetland complex of global ecological significance [22,23]. Nestled within the Wulianfeng Mountain range of the Wumeng Basin, the reserve spans 3150 hectares across altitudes of 3000–3200 m and features subalpine grasslands as its dominant landscape. Designated as a Ramsar Site in 2004 under the Convention on Wetlands of International Importance, it exemplifies global recognition for its unique peat marshes, alpine lakes, and role as a critical habitat for migratory waterbirds. This reserve not only sustains the endangered black-necked crane (*Grus nigricollis*), hosting over 1300 individuals during winter migrations, but also safeguards a fragile plateau ecosystem characterized by exceptional biodiversity [24]. Located in the upper reaches of the Jinsha River and the Hengjiang River watershed, it is a vital water conservation area in northeast Yunnan, China, renowned for its “water tower” effect and rich hydrological networks. Its diverse water systems, including alpine lakes, peat marshes, and riverine habitats, create ideal conditions for a wide range of aquatic organisms, particularly aquatic fungi, which play crucial roles in nutrient cycling and organic matter decomposition [25,26]. Likewise, they act as facilitators of carbon and nutrient transfer to invertebrates, fish, and zooplankton. They may contribute 20–40% to the zooplankton diet. Dashanbao Nature Reserve is a representative place to study aquatic fungi because of its favorable hydrological conditions, unique and multiple water environments, climatic conditions, geographic advantages, and special vegetation. However, research on the Reserve has mainly focused on the study of the black-necked crane [27], and little research has been devoted to its aquatic fungal resources.

The aim of this study was to examine the distribution and diversity of aquatic fungal communities in Dashanbao Nature Reserve and identify the relationship between fungi and environmental parameters. To the best of our knowledge, our study is the first to use next-generation sequencing methods to examine fungal communities in the Reserve. We provide new data for studying the diversity of fungi in the meadow wetland ecosystem of the subalpine marshy plateau, and we lay the groundwork for studies on the interactions between fungi and environmental factors.

## 2. Materials and Methods

### 2.1. Study Area and Sampling Methodology

To investigate the diversity and abundance of aquatic fungi in Dashanbao Nature Reserve, samples for high-throughput sequencing were collected on 16 October 2022 during the post-monsoon period. This timing was chosen to capitalize on stabilized hydrological conditions and peak fungal decomposition activity, which is driven by autumn litter input in subtropical high-altitude wetlands [28]. Starting from the Shida area (DS01), 12 sampling sites stretched across Dashanbao Nature Reserve (Table 1). The sampling stations were distributed throughout the site to encompass reservoirs, rivers, ponds, and marshes to obtain a thorough and accurate picture of the variety and distribution of fungi in the wetlands of the Reserve (Figure S1). To better understand how the surrounding environment affects water bodies locally, some focus was made on areas such as the concentration of tributaries, the edges of wetlands, and the outflow of reservoirs (Figure S2). The time, elevation, location, temperature, pH, water type, and other details of the sampling were documented to fully characterize the surrounding environment.

At all 12 sites, three replicate water samples (e.g., DS01_1, DS01_2, and DS01_3) were collected (at a 10 m horizontal interval) from a depth of 30 cm and on the same day. Each sampling location yielded about 20 L of water, which was used for genomic DNA extraction and the identification of chemical and physical indicators [29]. Surface water samples were collected by thoroughly shaking the buckets to ensure homogeneity, and approximately 1 L of water was filtered through a 0.45 μm membrane to capture suspended fungi. The filtration process concentrated fungal biomass onto the membrane, which was then carefully removed and used for subsequent DNA extraction [30].

### 2.2. DNA Extraction and Sequencing Analysis of Aquatic Fungi

The genomic DNA was extracted from the membrane filters of 36 water samples (1000 mL) using a PowerWater DNA Isolation Kit (MO BIO Laboratories, Inc., Carlsbad, CA, USA) according to the manufacturer’s instructions. The ITS2 region in the fungal DNA was amplified by PCR using the universal primers ITS1F (5’-CTTGGTCATTTAGAGGAAGTAA-3’) and ITS2R (5’-GCTGCGTTCTTCATCGATG C-3’). PCR was conducted using a 20 μL mixture containing 14 μL of sterile ddH_2_O, 2 μL of buffer (10×), 2 μL of dNTPs (2.5 mmol/L), 0.8 μL of each primer (5 μmol/L), 0.2 μL of 5U Taq DNA polymerase, 0.2 µL of BSA, and 10 ng of DNA template. The following reaction conditions were used: 95 °C for 3 min, 35 cycles at 95 °C for 30 s, annealing at 53 °C for 30 s, extension at 72 °C for 45 s, and 72 °C for 10 min. Using electrophoresis on a 2% agarose gel, the PCR products were determined. Using an Illumina MiSeq PE300 platform (Illumina, San Diego, CA, USA) [31], purified amplicons were pooled in equimolar and paired-end sequenced in accordance with the usual protocols by Majorbio Bio-Pharm Technology Co. Ltd. (Shanghai, China). The raw reads were deposited into the NCBI Sequence Read Archive (SRA) database under BioProject accession number PRJNA1205548 and under the Sequence Read Archive (SRA) accession numbers SRR12401455–SRR12401478.

To obtain high-quality and clean reads, Quantitative Insights Into Microbial Ecology (QIIME, http://qiime.org/install/index.html, accessed on 25 November 2023) was used for qualitative filtering of the raw sequences. FLASH was used to merge the two terminal sequences [32,33]. Then, the sequences were divided into operational taxonomic units (OTUs) based on a 97% similarity threshold value by using the clustering program VSEARCH (v1.9.6) [33]. The UNITE database (https://unite.ut.ee/, accessed on 25 November 2024) was used to identify the taxonomic diversity of the fungal communities [34].

### 2.3. Determination of Environmental Factors

We selected conventional water quality parameters, including pH, as baseline indicators for environmental characterization. Given the critical influence of nitrogen and phosphorus on algal dynamics and their sensitivity to nutrient availability, as well as the pivotal role of dissolved oxygen in aquatic ecosystem health, we additionally incorporated total nitrogen (TN), total phosphorus (TP), and dissolved oxygen (DO) as key analytical metrics. Field measurements of pH and DO were conducted using a multiparameter water quality analyzer (DZB-718L, LeiCi, Shanghai, China) [35]. For TN and TP quantification, a 10 L water sample was collected and processed in the laboratory to ensure accurate assessment of nutrient concentrations. Water samples were filtered (cellulose acetate filter, Ø 0.45 μm). TN were analyzed from the filtered water using a CN-Analyzer (multi N/C 3100, Analytik Jena GmbH, Jena, Germany) [36,37]. TP in water was extracted with the ascorbic acid ammonium molybdate method 34, and absorbance was measured at 700 nm [38].

### 2.4. Statistical Analyses

RStudio (Version 1.4.1717) was employed to compute the means and standard deviations of environmental factors. The alpha diversity of fungal communities was assessed using the “vegan” package in RStudio, employing the Shannon, Chao1, Simpson, and ACE indices [39]. Principal co-ordinate analysis (PCoA) was performed to evaluate the beta diversity of fungal communities using the Bray–Curtis dissimilarity matrix derived from OTU abundance data and implemented via the “ape”, “vegan”, and “ggplot2” packages in RStudio [39,40]. Redundancy analysis (RDA) was employed to explore the relationships between the horizontal community structure of fungal operational taxonomic units (OTUs) and four environmental factors in the sediments [41]. The FUNGuild database (http://www.funguild.org/, accessed on 30 October 2024) was used for function prediction [36]. Pearson’s correlation analysis was conducted in RStudio to examine correlations among fungal genera, trophic types, and environmental factors [39].

## 3. Results

### 3.1. Physicochemical Properties of Water Samples in Dashanbao Nature Reserve

Table 1 presents the geographic co-ordinates of 12 sampling sites across Dashanbao Nature Reserve, while Table 2 details the physicochemical properties of the water samples (Figure S3). The pH was within a narrow range of 7.8 to 9.2, slightly alkaline but within the limits of the China National Environmental Quality Standards for Surface Water. The concentration of DO varied greatly between sites, with concentrations as high as 11.1 mg/L at site DS08 and as low as 4.6 mg/L at Site DS01. The DO results were high at all points except DS01 and DS03.

TN was between 0.3243 and 7.8115 mg/L, with an average of 2.6596 mg/L. TP was between 0.0369 and 0.0427 mg/L, with an average of 0.0397 mg/L. Diurnal effects on TN/TP were negligible, as nutrient concentrations remained stable within the 6 h sampling window [42,43]. Analysis of variance showed that there were significant differences in TP content between different points (*p* < 0.01).

### 3.2. Composition and Distribution of Aquatic Fungi in Dashanbao Nature Reserve

We found 2,628,749 fungal ITS sequences clustered into 5829 OTUs with 97% sequence similarity (Table S1). A plateau appeared in each of the 12 rarefaction curves (Figure S4), with Good’s coverage values of 0.999 across all sites. It meant that the observed ITS sequences adequately represented all 12 sampling sites. The Chao1, ACE, Simpson, and Shannon indices indicated the difference in the abundance and diversity of fungi in the 12 sites (Table 3). Site DS05 exhibited the highest richness (Chao1 = 1710 ± 163, ACE = 1699 ± 184) and diversity (Shannon = 5.50 ± 0.00, Simpson = 0.0100 ± 0.0000), indicating a highly diverse and evenly distributed fungal community. In contrast, Sites DS09, DS10, and DS11 showed the lowest richness (Chao1 = 375 ± 33, 373 ± 29, 378 ± 13, respectively) and diversity (Shannon = 2.10 ± 0.35, 2.53 ± 0.38, 2.47 ± 0.50, respectively), suggesting less complex fungal assemblages. Sites DS01, DS03, DS06, DS07, DS08, and DS12 demonstrated moderate diversity, with Shannon indices ranging from 3.10 ± 0.72 to 4.53 ± 0.25. Notably, Simpson indices varied widely, from 0.0100 ± 0.0000 (DS05) to 0.2933 ± 0.0723 (DS09), reflecting differences in dominance patterns across sites. Overall, the data highlight distinct ecological niches within the reserve, with Site DS05 emerging as a potential hotspot for fungal biodiversity, while Sites DS09–DS11 represent areas of lower microbial complexity.

**Figure 1:**
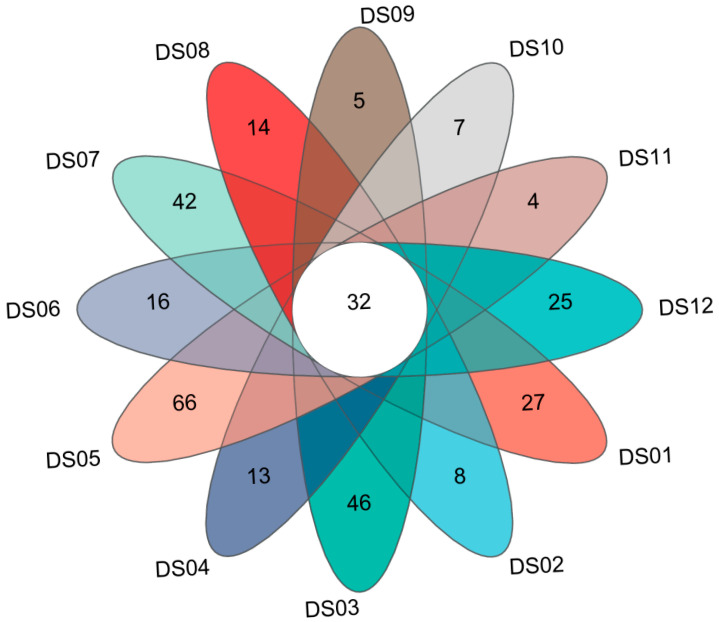
Venn diagram of OTUs for aquatic fungi from 12 sites (DS01–DS12) in Dashanbao Nature Reserve.

Among the 5829 OTUs of the aquatic fungi, there were 2337 OTUs that remained unclassified at the phylum level, and the remaining 3292 OTUs belonged to 649 genera in 16 phyla. The Venn diagram illustrates (Figure 1) the distribution of OTUs among 12 sampling sites, while the table presents the sequence reads and mean OTU numbers with standard deviations for each site (DS01–DS12) (Table 3). A total of 32 OTUs are shared among all 12 sites, indicating some common microbial groups. Each site also has unique OTUs, highlighting the site-specific microbial compositions. There are significant differences in the number of OTUs among sites, with DS05 having the highest mean number (1455 ± 108) and DS09 the lowest (278 ± 40).

**Figure 2:**
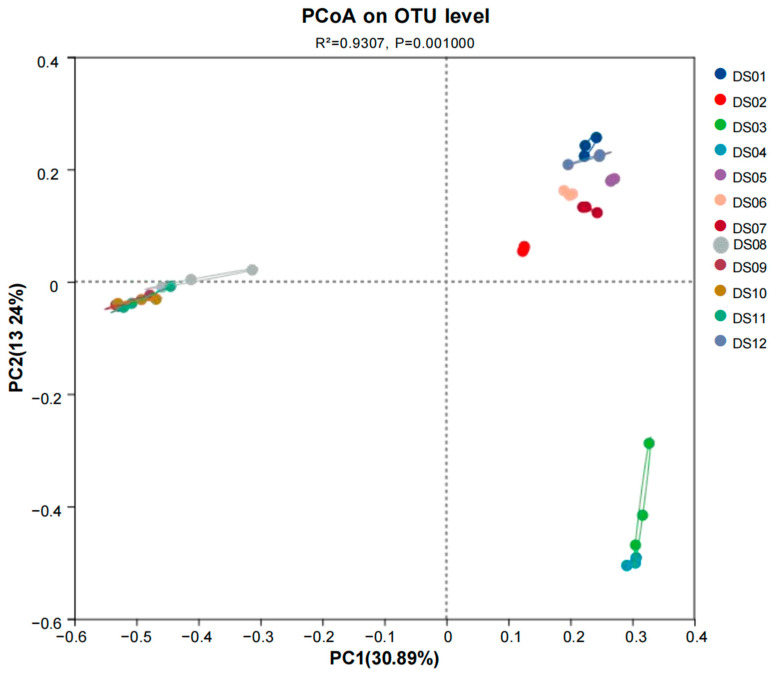
Principal co-ordinate analysis of OTUs from 12 sites in Dashanbao Nature Reserve wetland.

Principal co-ordinate analysis (PCoA) was employed to delve into the composition of fungal communities at the OTU level across the 12 sampling sites, offering insights into the similarities and differences in their microbial profiles. As depicted in the PCoA plot (Figure 2), the subsamples from DS04 and DS08–DS11 were scattered in the ordination space, indicating a relatively high degree of dissimilarity among them in terms of OTU composition. This implies that the environmental conditions or other factors at these sites may have led to distinct fungal communities. Conversely, the subsamples from DS01, DS05, DS06, DS07, and DS12 clustered closely together, suggesting a significant similarity in their fungal community compositions. These sites might share common environmental characteristics that promote the growth of similar sets of OTUs. The subsamples from DS03 and DS04 exhibited a clear aggregation with each other but were positioned far from the other sites. This indicates that these two sites have a unique combination of factors that result in a fungal community structure distinct from the rest. The samples from Site DS02 were relatively isolated in the plot, highlighting their independent nature in terms of community composition on OTU level. It could be that specific local conditions at DS02, such as unique substrate availability or microclimate, have shaped a separate fungal community. Finally, some of the samples from DS08 to DS11 formed an aggregated group. While they are somewhat similar to each other, their position in the plot also shows that they are different from the other major clusters, suggesting a moderately distinct fungal community structure within this group of sites.

**Figure 3:**
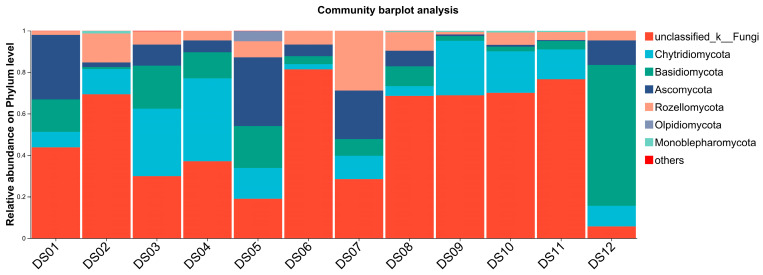
The aquatic fungal composition and distribution of 36 samples at phylum level from 12 sites (DS01–DS12) in Dashanbao Nature Reserve. ‘Others’ combines 12 phyla each contributing <1% abundance.

At the phylum level, Ascomycota (51.75%) was the dominant group of aquatic fungi, followed by Chytridiomycota (20.30%), Basidiomycota (19.33%), and Rozellomycota (6.47%). The remaining 12 phyla collectively accounted for less than 1% of the total abundance (Table S2). Specifically, the aquatic fungal communities across the 12 sampling sites in Dashanbao Nature Reserve exhibited both shared and distinct characteristics at the phylum level (Figure 3). Ascomycota was the dominant phylum, consistently representing the highest relative abundance across most sites, which aligns with its well-known role as a major decomposer in aquatic ecosystems. Chytridiomycota and Basidiomycota were also prevalent, though their relative abundances varied more significantly between sites, suggesting niche-specific adaptations to local environmental conditions. Notably, *Rozellomycota*, while present at all sites, showed lower and more variable abundance (6.47%), indicating its sensitivity to site-specific factors such as nutrient availability or pH. Collectively, the other 12 minor phyla (e.g., *Mortierellomycota*, *Glomeromycota*) represented less than 1% of total abundance, with individual contributions below 0.3%. Sites with higher overall diversity, such as DS05, displayed a more even distribution of dominant phyla, while sites with lower diversity, like DS09–DS11, were characterized by a stronger dominance of Ascomycota (78.2%) and reduced representation of minor phyla.

For the classified genera, *Tausonia* (4.17%), *Cladosporium* (1.89%), *Leucosporidium* (1.42%), and *Cystofilobasidium* (1.05%) were the top four genera, and approximately 73.25% of the sequences belonged to unclassified fungi (Figure S2). The community structure of aquatic fungi in the 12 sites (DS01–DS12) shows both similarities and differences (Figure S5). There are several common genera, such as *Cladosporium*, *Tausonia*, *Vishniacozyma*, *Cystofilobasidium*, and *Filobasidium*, which are widely distributed across multiple samples. However, their abundances vary significantly among different sites. For example, *Cladosporium* accounts for 7.59% in DS01 but only 0.26% in DS09. In terms of dominant genera, each sample has its own unique composition. Notably, DS01 is characterized by a relatively high abundance of *Cladosporium* (7.59%), *Tausonia* (3.44%), and *Simplicillium* (3.26%), suggesting strong adaptability of these genera in this environment. In contrast, DS12 stands out with *Tausonia* being the predominant genus (42.66%), followed by *Leucosporidium* (8.77%) and *Cutaneotrichosporon* (6.26%), indicating a unique fungal community composition compared to other sites. Moreover, in some sites like DS02, DS04, DS06, DS09, DS10, and DS11, the proportion of unidentified genera (others) is extremely high, suggesting that there may be a large number of unclassified or low-abundance fungal taxa that need further exploration. Overall, these results indicate that the environmental conditions of each sampling site play a crucial role in shaping the community structure of aquatic fungi, and further research on the ecological functions of these fungi and their relationships with environmental factors is warranted.

### 3.3. Function Prediction of Fungi by FUNGuild

**Figure 4:**
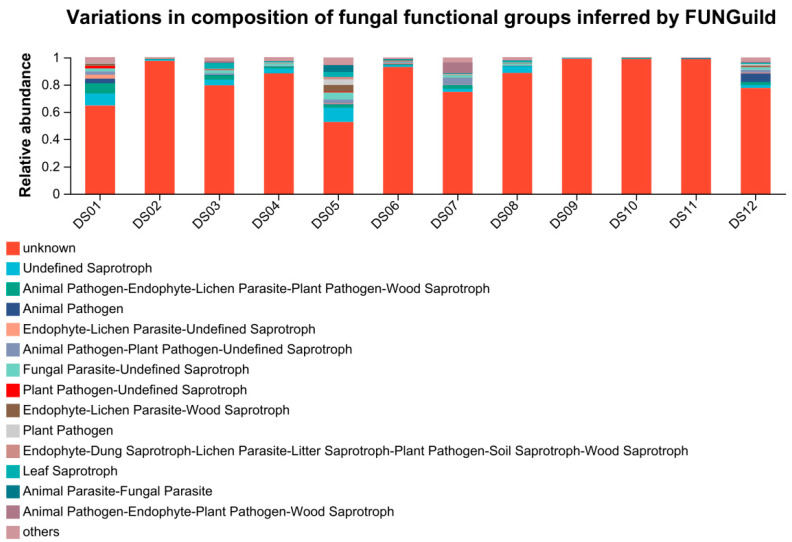
Distribution of fungal trophic types at 12 sites in Dashanbao Nature Reserve on genus level based on FUNGuild platform.

Using the FUNGuilD platform, the nutritional and 299 functional groups of fungal communities in different samples were predicted. Functional prediction classified aquatic fungi into 13 major trophic modes after excluding low-abundance taxa (<0.01%) and unassignable groups (Table S3). These functional groups included saprotroph (12.4%), pathotroph-saprotroph-symbiotroph (5.4%), pathotroph (4.5%), pathotroph-saprotroph (4.3%), symbiotroph (1.4%), saprotroph-symbiotroph (1.2%), pathotroph-symbiotroph (0.7%), and pathogen-saprotroph-symbiotroph (0.2%). Significant differences existed among the sampling sites in the composition of fungal functional groups. Detailed information on the fungal trophic types and their distribution can be found in Figure 4.

### 3.4. Correlation Between Fungi and Environmental Factors

**Figure 5:**
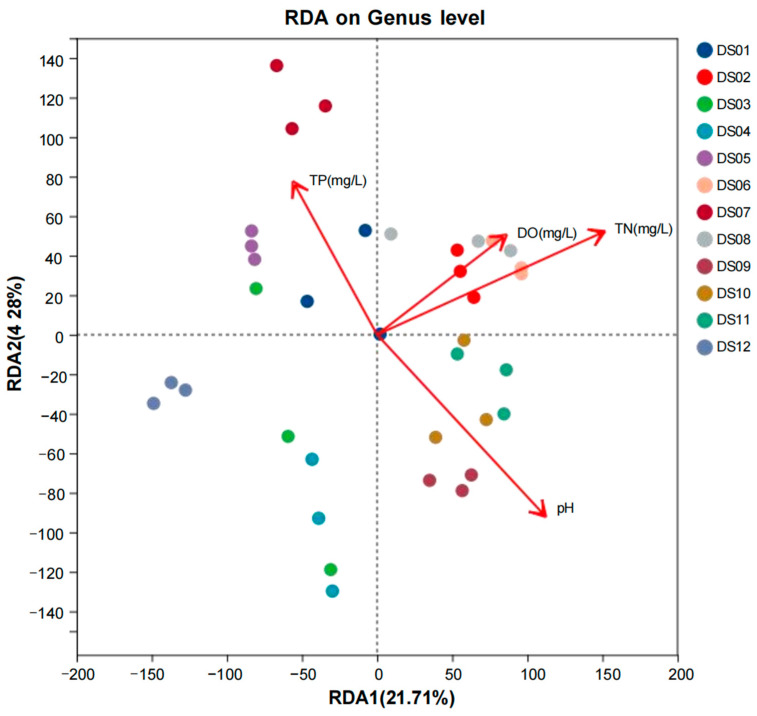
Redundancy analysis of fungal community structure and environmental factors at 12 sites in Dashanbao Nature Reserve. TN stands for total nitrogen, TP stands for total phosphorus, and DO stands for dissolved oxygen.

To explore the relationship between aquatic fungal communities and their environment, redundancy analysis (RDA) was performed on the horizontal community structure of aquatic fungi OTUs in relation to four environmental factors, as depicted in Figure 5. The first and second ordination axes explained 21.71% and 4.28% of the variation in the dataset, respectively. Further analysis revealed that the environmental factors significantly influence the distribution of fungal communities. Sampling sites like DS02 and DS06, which are relatively close to the direction of the TN arrow, suggest that an increase in TN concentration may be associated with a certain composition of the fungal community in these sites. Higher TN levels might enhance the growth of specific fungal genera that are adapted to utilize nitrogen-rich resources, potentially leading to an increase in their relative abundance within the community. For TP, sampling sites such as DS01, DS03, and DS05 show a tendency to align with the direction of the TP arrow. This indicates that an increase in TP concentration may create a favorable environment for specific fungal genera within these sites. These fungi might have metabolic pathways adapted to utilize phosphorus, and higher TP levels could potentially boost their growth and relative abundance, thus shaping the composition of the fungal community in areas with elevated TP. Regarding pH, the arrow direction of pH shows an opposite trend to certain sampling sites, like DS09 and adjacent points. Fungal genera in sites closer to the pH arrow may have specific physiological tolerances to the corresponding pH conditions. In summary, our findings indicate that TN, TP, and pH are the predominant environmental factors that shape the structure and composition of fungal communities in this aquatic ecosystem. These factors not only directly influence fungal growth and distribution but also interact with each other in complex ways, creating a dynamic environment that determines the ecological niches and community assembly of aquatic fungi.

**Figure 6:**
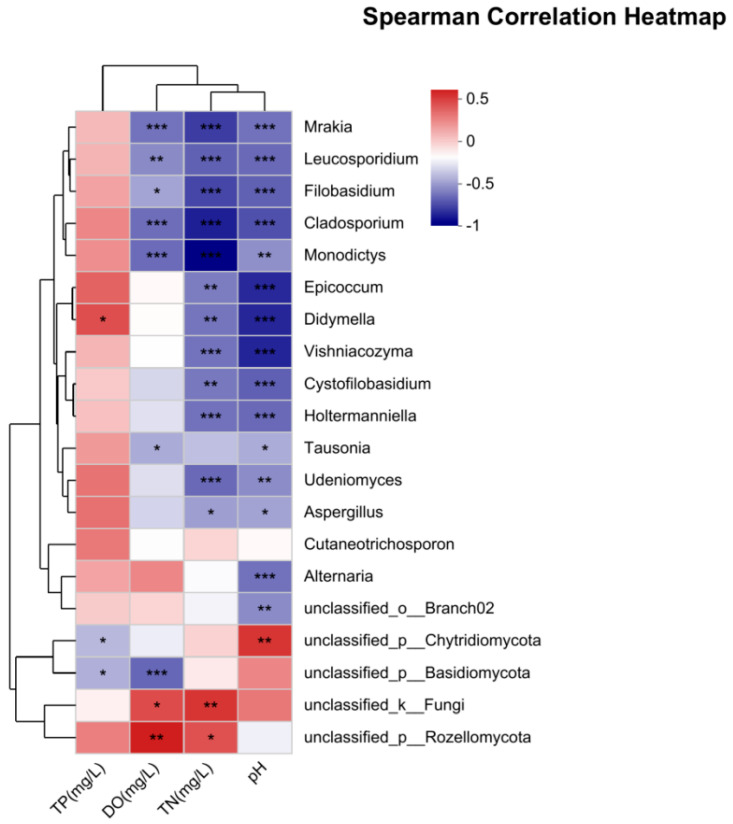
Heatmap of correlation between fungal community composition (top 20) and environmental factors of taxonomic genera. * indicates significant difference at *p* < 0.05; ** indicates extremely significant difference at *p* < 0.01; *** indicates highly significant difference at *p* < 0.001.

Principal component analysis (PCA) revealed that, among the top 20 fungal genera, the compositions of 14 genera were significantly correlated with environmental factors, with the exclusion of five unidentified ones (Figure 6). These genera were *Mrakia*, *Leucosporidium*, *Filobasidium*, *Cladosporium*, *Monodictys*, *Epicoccum*, *Didymella*, *Vishniacozyma*, *Cystofilobasidium*, *Holtermanniella*, *Tausonia*, *Udeniomyces*, *Aspergillus*, and *Alternaria.* All of these genera had a significant negative correlation with pH. Apart from *Tausonia*, *Cutaneotrichosporon*, and *Alternari*, the other 12 identified genera were significantly negatively correlated with TN. *Mrakia*, *Leucosporidium*, *Filobasidium*, *Cladosporium*, *Monodictys*, and *Tausonia* were negatively correlated with DO. *Didymella* was significantly positively correlated with TP.

### 3.5. Correlation Analysis Between Trophic Modes and Environmental Factors

**Figure 7:**
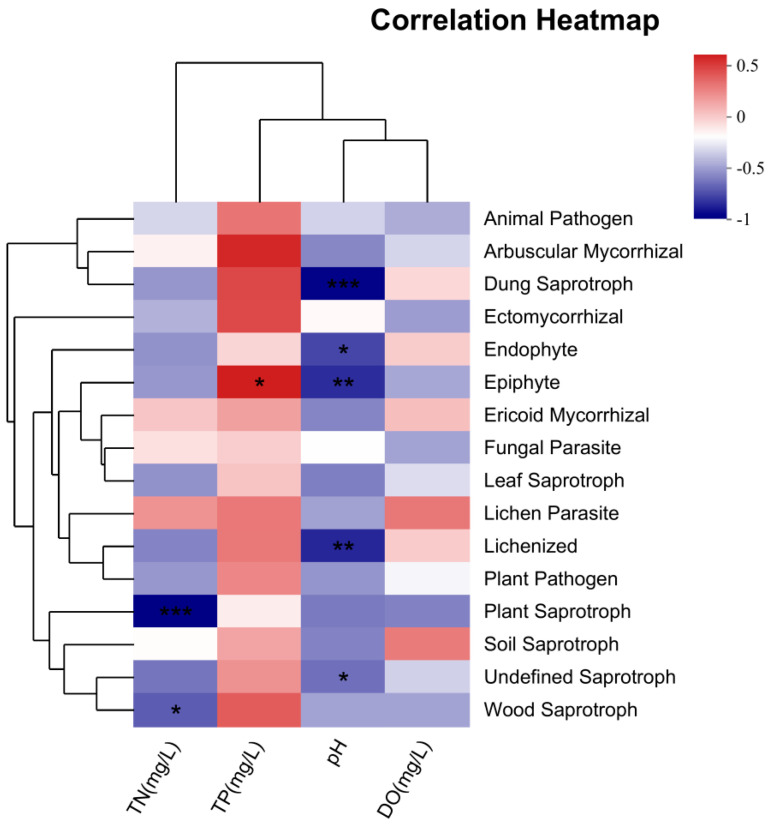
Heatmap of correlations between fungal trophic mode composition (top 16) and environmental factors. * indicates significant difference at *p* < 0.05; ** indicates extremely significant difference at *p* < 0.01; *** indicates highly significant difference at *p* < 0.001.

According to a heatmap (Figure 7), four environmental factors and trophic modes were correlated. Specifically, plant saprotrophs and wood saprotrophs had a significant negative correlation with TN. Epiphyte showed a significant positive correlation with TP. DO did not show any significant correlation with the trophic modes. Dung saprotrophs, endophyte, epiphyte, and lichenized fungi had highly negative correlations with pH.

## 4. Discussion

As an integral part of the aquatic network, aquatic fungi are crucial to the material cycle and energy flow of freshwater ecosystems. Thus, it is necessary to establish the fungal diversity and its relationship to environmental factors. Nestled within Dashanbao Nature Reserve, a globally significant wetland ecosystem renowned for its rich biodiversity and unique geographical features, we identified an ideal study site for investigating aquatic fungi. This research explored the effects of environmental factors on the diversity and composition of aquatic fungi in the Reserve.

### 4.1. Environmental Factors in Dashanbao Nature Reserve Water

There were some parallels and variances in the environmental variables of the locations (Table 2 and Figure S3). The pH was slightly alkaline but remained within the regulated levels stipulated by China National Environmental Quality Standards for Surface Water. Although the small changes in pH were not likely to have directly impacted aquatic life, they greatly influence the horizontal community structure [44,45]; this fact was supported by the results of the redundancy analysis. In addition, the availability and solubility of all chemical forms in the lake may affect nutrients. A low pH may increase the solubility of phosphorus, making it more available for plant growth and resulting in a greater long-term demand for DO [46]. Consequently, it is imperative to pay close attention to the variation in pH.

DO levels were high at all points except for DS01 and DS03. The factor directly reflects the balance between respiration, photosynthesis, and decomposition, i.e., it is metabolism-related [47]. Furthermore, DO directly affects aquatic fauna by its impact on animal behavior and survival [48,49]. Thus, DO content has an important impact on aquatic life survival and water quality [50]. High concentrations of microorganisms and aquatic plants (algae) will reduce the amount of DO in the water body, which indicates relatively good water quality. The activity and make-up of fungal communities can be influenced by concurrent changes in oxygen and nutrient availability in aquatic settings [51]. DO was lowest at DS01 and DS03, which might have been due to rapidly growing aquatic plants that consume large amounts of oxygen in the autumn.

Both TN and TP surpassed the 0.2 mg/L and 0.02 mg/L limits, respectively, which are set by China National Environmental Quality Standards For Surface Water. The possible cause of this excess could be the rural characteristics of these areas [52,53]. Nitrogen and phosphorus, as crucial nutrients, have diverse forms that play a significant role in evaluating how aquatic ecosystems react to environmental alterations and the influence of human activities [54,55]. These elements can trigger blooms of microorganisms and aquatic plants, which may potentially result in eutrophication [56]. A significant correlation has been found between the chlorophyll in stream benthos and the levels of TN and TP in the water column. Combined, these nutrients account for a greater amount of variance than either one alone, which is consistent with the research of Dodds et al. [57]. In the investigation and statistics of this study, TN and TP concentrations varied significantly (Table 3). In the majority of the areas under study, the levels of nitrogen and phosphorus were found to be high, particularly at Sites DS02, DS07, and DS08. Conversely, relatively low concentrations of TN and TP were detected at Sites DS01, DS04, and DS05. It is hypothesized that the higher concentrations at DS02, DS07, and DS08 may be attributed to the grazing and planting activities proximal to the wetland areas in the vicinity of these sites.

### 4.2. Fungal Diversity and Distribution

Chao1, ACE, Shannon, and Simpson offer distinct perspectives on the fungal community characteristics at the 12 sites in Dashanbao Nature Reserve. Compared with previous studies on fungal diversity in freshwater [58], the fungal diversity in the Dashanbao Nature Reserve was high, which might be caused by the different environments. The pronounced heterogeneity in fungal diversity across wetlands in Dashanbao Nature Reserve—ranging from highly diverse alpine lakes (DS05: Shannon = 5.50) to depauperate reservoirs (DS11: Shannon = 2.47)—primarily reflects niche partitioning driven by environmental complexity. Specifically, hydrological connectivity between peat marshes and river networks facilitates microbial dispersal [59], while allochthonous organic inputs from subalpine grasslands sustain diverse saprotroph guilds through pulsed resource availability [60]. Concurrently, thermal stratification in deep reservoirs creates microhabitats favoring psychrophilic Chytridiomycota [61], collectively explaining the dominance of sediment-associated Ascomycota in decomposition hotspots versus planktonic Basidiomycota in oligotrophic sites. This environmental mediation of community structure contrasts sharply with homogeneous fungal assemblages in low-complexity aquatic ecosystems [58], underscoring how the reserve’s habitat mosaicism maintains exceptional microbial biodiversity. Compared to other high-altitude wetlands (>2600 masl), Shannon diversity (2.10–5.50) exceeded Himalayan peat bogs (Shannon ≤ 1.81) [62,63], but was lower than Andean lakes (4.1–6.7) [64,65], reflecting its unique mix of oligotrophic and nutrient-impacted sites.

A total of 649 genera belonging to 16 phyla were identified in the water of Dashanbao Nature Reserve. At the phylum level, Ascomycota accounted for 51.75%, followed by Chytridiomycota (20.30%), Basidiomycota (19.33%), and Rozellomycota (6.47%). Numerous studies have identified these phyla within aquatic ecosystems. For example, Rojas-Jimenez et al. [66] found that Cryptomycota and Chytridiomycota were the most dominant fungal phyla in their study of fungal diversity within ice-covered lakes of the McMurdo Dry Valleys in Antarctica. Debeljak and Baltar [64] determined that the most abundant taxa in terrestrial freshwater and marine ecosystems were Ascomycota and Basidiomycota, with the exception of freshwater rivers, where Chytridiomycota was the dominant phylum. In a comprehensive analysis by Jiya et al. [66], exploring fungal diversity and distribution across various niches including exposed soil, snow accumulation, deepsea, and lake sediments in the Larsemann Hills, they found Ascomycota to be the most dominant phylum at 61.7%, followed by Basidiomycota (31.1%), Chytridiomycota (5.7%), and Rozellomycota (1.4%) [62].

At almost every site, 32 fungal genera were the most abundant, with different proportions. The Site DS05 owned the most unique OTUs, about 66 OTUs, a site with good water quality consistent with previous studies [67,68]. Fungi are gaining worldwide attention as promising biological indicators for defining the trophic status of riverine systems. Fungal genera such as *Aspergillus*, *Kluveromyces*, *Lodderomyces*, *Nakaseomyces*, and *Penicillium* are potential bioindicators of river pollution and eutrophication [69]. A further issue of note is the distinct composition of the dominant fungi at the DS12 site, which contrasts sharply with the other sites, being predominantly *Tausonia* (42.66%). *Tausonia* is a human pathogen basidiomycetous yeast of the *Cystoflobasidiales* order [70,71]. Recent research on the nonpoint source pollution (NPSP) in the Jialing River basin shows that NPSP significantly increased the relative abundance of *Tausonia* among sediment fungi, suggesting its potential as an indicator of ecological changes in the river’s aquatic environment due to NPSP [72]. In the study of hypersaline soils in the Urmia Lake National Park, Iran, *Tausonia* was among the identified basidiomycetous yeast genera [73]. Strains belonging to the genus have biotechnological potential, including the production of cold-active enzymes, such as pectinolytic and β-galactosidase [74,75]. Therefore, the potential for the dominant aquatic fungi in Dashanbao Nature Reserve remains to be examined, including potential biological indicators for global environmental changes [76].

In this study, 2337 OTUs (40.1% of total OTUs) remained unclassified at the phylum level, while an even more substantial gap was observed at the genus level, with 73.25% of sequences taxonomically unresolved, collectively highlighting significant limitations in existing fungal reference databases for high-altitude wetlands. This pervasive taxonomic uncertainty likely stems from two interrelated factors: first, inherent database biases as exemplified by UNITE’s primary coverage of well-characterized ecosystems [34]. Second, the probable existence of novel phylogenetic lineages uniquely adapted to Dashanbao’s extreme conditions—including persistent low temperatures, high UV radiation, and dynamic hydrological regimes. Crucially, similar patterns of microbial “dark matter” have been documented in analogous understudied ecosystems such as Antarctic ice-covered lakes and deep-sea sediments [62,66], where cryptic taxa frequently encode essential functional traits for local biogeochemical cycling. Consequently, resolving these taxonomic voids necessitates expanded genomic resources complemented by culture-dependent approaches; future research integrating metagenome-assembled genomes with targeted isolation strategies promises to elucidate the specific contributions of these enigmatic fungi to organic matter decomposition and nutrient dynamics in alpine wetlands, thereby advancing our understanding of microbial diversity conservation in globally significant Ramsar sites.

### 4.3. Correlation Analysis Between the Fungal Communities and Environmental Factors

The redundancy analysis (RDA) on the genus level illustrated the relationships between environmental factors and fungal community samples. Visually, the sample points were scattered across the graph, indicating significant variations in the distribution of aquatic fungi among different sampling sites. Conversely, TN-driven negative correlations (r = −0.67 avg.) indicate nitrogen inhibition of ligninolytic enzymes; elevated TN represses laccase/peroxidase expression in basidiomycetes [77], explaining *Filobasidium*’s decline (r = −0.76). The TP-*Didymella* positive linkage (r = +0.71) aligns with its phosphorus-dependent pathogenicity—high TP enhances host plant cell wall degradation via polygalacturonase induction [78]. The robust pH-genus correlations (mean r = −0.73) reflect membrane fluidity adaptations, where acid-tolerant fungi maintain optimal proton gradients through increased unsaturated fatty acids in cell membranes. The study by Hu et al. [79] demonstrated that elevated TN and TP levels significantly influenced microbial community structure and cyanobacterial blooms in a freshwater aquaculture pond, highlighting the critical role of nutrient overloading in shaping aquatic ecosystems. The study by Tian et al. [80] identified TN as a key environmental factor significantly influencing the structure of fungal communities in the lakes of the Headwater Region of the Yellow River in China. This finding underscores the importance of nutrient availability, particularly nitrogen, in shaping microbial community composition in aquatic ecosystems. The study by Wu et al. [39] found DO and pH significantly influenced the distribution of aquatic fungal genera, with *Aspergillus* and *Didymosphaeria* showing positive correlations with DO, while *Laetisaria*, *Fusarium*, and *Phoma* exhibited significant negative correlations with pH, highlighting the critical roles of DO and pH in shaping fungal community composition in aquatic environments. The research by Lin et al. [81] also demonstrated that pH and nitrogen levels significantly shaped the diversity and distribution of fungal communities in a river, emphasizing their importance in structuring fungal assemblages and their ecological roles in aquatic environments.

The PCA results revealed that the compositions of 14 out of the top 20 fungal genera were significantly influenced by environmental factors, underscoring the importance of pH, TN, DO, and TP in shaping fungal community structure in the studied ecosystem. Strikingly, all 14 genera-including psychrophilic yeasts (*Mrakia*, *Leucosporidium*) and decomposers (*Cladosporium*, *Monodictys*) showed pronounced negative correlations with pH (r = −0.62 to −0.83, *p* < 0.01), indicating specialized adaptations to acidic conditions through membrane lipid saturation and enhanced proton extrusion [82]. Concurrently, nitrogen enrichment inhibited 12 genera (excluding *Tausonia* and *Alternaria*), most notably suppressing ligninolytic *Filobasidium* (r = −0.76) via TN-induced repression of laccase transcription [83], while hypoxia preferentially selected fermentative specialists like *Leucosporidium* (r = −0.68) through pyruvate decarboxylase activation that bypasses oxidative phosphorylation [84]. Conversely, *Didymella* thrived under phosphorus enrichment (r = +0.71), where TP-dependent polygalacturonase induction enhanced plant cell wall deconstruction [85], illustrating how nutrient–physiology interactions create ecological trade-offs: TP enrichment favored pathogens but displaced oligotrophic specialists (*Mrakia* pH r = −0.83/TN r = −0.75), thereby mediating stoichiometric niche partitioning across the wetland gradient. Totally, all 14 identified genera, including *Mrakia*, *Leucosporidium*, *Filobasidium*, *Cladosporium*, *Monodictys*, *Epicoccum*, *Didymella*, *Vishniacozyma*, *Cystofilobasidium*, *Holtermanniella*, *Tausonia*, *Udeniomyces*, *Aspergillus*, and *Alternaria*, exhibited significant negative correlations with pH. This suggests that these genera may thrive in environments with lower pH levels, potentially due to their physiological adaptations to acidic conditions. Such findings align with previous studies demonstrating that pH is a critical factor shaping fungal community composition, as it directly affects nutrient availability and enzymatic activities [81]. Furthermore, 11 of these genera (excluding *Tausonia*, *Cutaneotrichosporon*, and *Alternaria*) showed significant negative correlations with total TN, indicating that increased nitrogen levels may inhibit their growth or favor competing taxa. This observation is consistent with the known sensitivity of many fungi to nitrogen availability, which can influence their metabolic processes and competitive interactions [86]. Interestingly, *Mrakia*, *Leucosporidium*, *Filobasidium*, *Cladosporium*, *Monodictys*, and *Tausonia* were negatively correlated with DO, suggesting a preference for low-oxygen conditions, consistent with findings that certain fungi, like *Articulospora tetracladia*, thrive under reduced oxygen levels [87]. In contrast, *Didymella* displayed a significant positive correlation with TP, highlighting its potential role in phosphorus-rich environments. This genus is known for its saprotrophic capabilities and may thrive in conditions where phosphorus availability supports organic matter decomposition and nutrient cycling [85].

Overall, these findings underscore the intricate interactions between environmental factors and fungal community composition. The robust correlations detected between particular fungal genera and pH, TN, DO, and TP indicate that these elements serve as pivotal drivers of fungal distribution and ecological function within the studied ecosystem. For instance, the significant relationships imply that changes in these environmental factors can directly impact the presence, abundance, and activities of specific fungal genera, thereby influencing the overall structure and function of the fungal community. However, the divergent responses of certain genera, such as *Cladosporium*, to environmental variables like DO bring to light the necessity for further research. Elucidating the underlying mechanisms and potential synergistic effects of multiple environmental factors on fungal communities is crucial for a more comprehensive understanding. Moreover, aside from the differences in fungal structure induced by the environmental factors we investigated, factors like population growth and breeding, which can disrupt the wetland environment, also contribute to variations in fungal diversity, as indicated by previous studies [88,89].

### 4.4. Correlation Between Fungal Function Prediction and Environmental Factors

The FUNGuild analysis indicated that saprotrophs were a major trophic mode among the aquatic fungi at the 12 sites in Dashanbao Nature Reserve, aligning with previous research showing their prevalence in aquatic ecosystems [39]. This dominance underscores their crucial role in the ecosystem. Saprotrophs, being vital decomposers, play a fundamental part in nutrient cycling by consuming nutrients through the degradation of apoptotic cells [90]. As revealed by Wang et al. [91], fungal community dynamics, especially those of saprotrophs, are significantly influenced by the two factors. High levels of TN might lead to imbalances in the nutrient-acquisition process. For example, an excess of nitrogen could potentially stimulate the growth of other microorganisms that compete with saprotrophs for essential carbon sources, thus affecting the growth and abundance of saprotrophs [92]. However, plant saprotrophs and wood saprotrophs showed a significant negative correlation with TN in our study, which may reflect their adaptation to low-nitrogen environments. These fungi are often involved in decomposing complex plant polymers like cellulose and lignin, processes that are less dependent on nitrogen availability. Additionally, the negative correlation with TN suggests that high nitrogen levels might favor other microbial groups, reducing the competitive advantage of these saprotrophs.

Epiphytes exhibited a significant positive correlation with TP, indicating that phosphorus availability may enhance their growth and colonization of plant surfaces. Phosphorus is a critical nutrient for many fungi, and its abundance likely supports the metabolic demands of epiphytic fungi, which often inhabit nutrient-limited environments such as plant surfaces. This correlation aligns with studies showing that phosphorus enrichment can promote the growth of epiphytic communities in aquatic ecosystems.

DO did not show significant correlations with any trophic modes, which may reflect the adaptability of aquatic fungi to varying oxygen levels. Some aquatic fungi are facultative anaerobes, capable of thriving in both oxygen-rich and oxygen-depleted environments [87], which could explain the lack of a clear relationship with DO. Alternatively, the influence of DO might be masked by the effects of other co-varying environmental factors, such as pH or nutrient availability.

### 4.5. Limitations and Future Perspectives

In the study, we focused on aquatic fungal diversity in surface water with a single-depth sampling at 30 cm, which may introduce potential sampling bias. This single-depth approach could either overrepresent or underrepresent the functional diversity of fungi across the entire water column, as fungal communities might vary significantly with depth due to differences in light penetration, oxygen levels, nutrient distribution, and substrate availability. To address this constraint in future research, we propose implementing a multi-depth sampling strategy that covers different layers of the water column. This approach would provide a more holistic understanding of aquatic fungal diversity and its distribution patterns across vertical gradients. Additionally, integrating environmental factor measurements at each corresponding depth would enable a more comprehensive analysis of the relationship between fungal diversity and specific environmental variables within different water layers in Dashanbao Nature Reserve.

## 5. Conclusions

This study provides the first comprehensive analysis of aquatic fungal communities in surface waters (30 cm depth) of Yunnan Dashanbao Black-necked Crane National Nature Reserve, a Ramsar wetland of global significance in China. We demonstrate that this high-altitude ecosystem harbors substantial fungal diversity, with 5829 OTUs spanning 15 phyla dominated by Ascomycota. Notably, site-specific richness variations were observed, indicating strong microenvironmental filtering within the reserve. Furthermore, our analyses identified total nitrogen (TN) and pH as primary determinants of community structure. TN emerged as the strongest driver, exhibiting significant negative correlations with 11 fungal genera, while pH influenced 14 genera with particularly strong negative associations in taxa like Mrakia and Cladosporium. Functionally, saprotrophs constituted the dominant trophic guild, with plant and wood saprotrophs showing pronounced negative correlations with TN. This nutrient sensitivity suggests stoichiometric regulation of decomposition processes, where elevated nitrogen may suppress certain organic matter degradation pathways. Looking forward, future studies should implement multi-depth sampling to resolve vertical stratification of fungal communities and expand genomic resources for the substantial proportion of unclassified taxa, which may represent novel lineages adapted to extreme alpine conditions.

## Figures and Tables

**Table 1:** Basic information of 12 sites collected from Dashanbao Nature Reserve.

Site	GPSN (Latitude)	GPSE (Longitude)	Elevation (m)
DS01	27°25′21″	103°21′54″	2887
DS02	27°27′59″	103°22′28″	2841
DS03	27°27′39″	103°21′51″	2684
DS04	27°28′50″	103°21′32″	2925
DS05	27°28′30″	103°21′21″	2708
DS06	27°27′20″	103°19′15″	2708
DS07	27°26′28″	103°19′23″	3002
DS08	27°25′39″	103°18′13″	3015
DS09	27°23′35″	103°16′15″	3020
DS10	27°23′30″	103°17′44″	3147
DS11	27°22′54″	103°19′13″	3125
DS12	27°20′20″	103°60′54″	2742

**Table 2:** Physicochemical properties of water from 12 sites in Dashanbao Nature Reserve.

Site	Total Nitrogen (mg/L)	Total Phosphorus (mg/L)	pH	Dissolved Oxygen (mg/L)	Sampling Time
DS01	0.3982 ± 0.0271 a	0.0404 ± 0.0018 abc	7.9 ± 0.3 a	4.6 ± 0.3 a	08:00
DS02	7.8115 ± 0.3462 g	0.0427 ± 0.0029 c	9.2 ± 0.2 c	12.0 ± 0.3 g	08:15
DS03	1.6577 ± 0.1676 c	0.0424 ± 0.0062 bc	8.0 ± 0.3 a	7.2 ± 0.3 b	09:30
DS04	0.9654 ± 0.2035 b	0.0388 ± 0.0012 abc	8.0 ± 0.2 a	10.0 ± 0.3 e	10:00
DS05	0.3243 ± 0.0222 a	0.0392 ± 0.0007 abc	7.9 ± 0.2 a	9.0 ± 0.3 c	11:20
DS06	1.0679 ± 0.0588 b	0.0400 ± 0.0012 abc	7.8 ± 0.3 a	10.0 ± 0.3 e	12:30
DS07	2.8115 ± 0.1387 d	0.0408 ± 0.0034 abc	7.8 ± 0.1 a	10.8 ± 0.3 f	13:15
DS08	5.8500 ± 0.0666 f	0.0385 ± 0.0024 abc	8.0 ± 0.2 a	11.1 ± 0.3 f	13:45
DS09	2.6192 ± 0.0385 d	0.0373 ± 0.0007 ab	9.1 ± 0.2 c	9.5 ± 0.2 d	14:00
DS10	2.7987 ± 0.0222 d	0.0369 ± 0.0007 a	8.5 ± 0.1 b	9.4 ± 0.1 cd	14:30
DS11	4.1321 ± 0.0444 e	0.0392 ± 0.0018 abc	8.7 ± 0.2 b	9.4 ± 0.2 cd	15:15
DS12	1.4782 ± 0.0222 c	0.0402 ± 0.0032 abc	8.4 ± 0.1 b	9.4 ± 0.2 cd	15:45

Note: The different lowercase letters indicate that the difference was significant at the 0.05 level; n = 3.

**Table 3:** Fungal sequences, operational taxonomic unit (OTU) richness, and diversity indices of ITS sequences for clustering at 97% similarity.

Site	Sequence Number	OTUs	Chao1	ACE	Shannon	Simpson
DS01	61412 ± 3575 a	741 ± 108 b	849 ± 132 b	842 ± 141 b	4.53 ± 0.25 ab	0.0333 ± 0.0116 ab
DS02	78279 ± 4075 cde	371 ± 42 a	421 ± 45 a	421 ± 60 a	2.87 ± 0.21 bcde	0.1400 ± 0.0265 bc
DS03	76049 ± 1396 cd	766 ± 40 b	879 ± 47 b	871 ± 53 b	4.20 ± 0.26 ab	0.0633 ± 0.0252 ab
DS04	61069 ± 11892 a	377 ± 22 a	407 ± 39 a	402 ± 37 a	3.37 ± 0.25 abcd	0.1133 ± 0.0208 abc
DS05	62240 ± 7667 a	1455 ± 108 c	1710 ± 163 d	1699 ± 184 e	5.50 ± 0.00 a	0.0100 ± 0.0000 a
DS06	69028 ± 5308 abc	727 ± 64 b	1065 ± 52 c	1060 ± 64 c	2.77 ± 0.29 def	0.2300 ± 0.0346 cd
DS07	75022 ± 8635 bcd	706 ± 71 b	833 ± 84 b	828 ± 68 b	3.77 ± 0.21 abc	0.0800 ± 0.0173 ab
DS08	76078 ± 1947 cd	700 ± 61 b	862 ± 59 b	863 ± 47 b	3.27 ± 0.59 def	0.2200 ± 0.1015 cd
DS09	81796 ± 1229 de	278 ± 40 a	375 ± 33 a	366 ± 8 a	2.10 ± 0.35 f	0.2933 ± 0.0723 d
DS10	81475 ± 1563 de	310 ± 36 a	373 ± 29 a	373 ± 13 a	2.53 ± 0.38 def	0.2267 ± 0.0808 cd
DS11	89872 ± 5679 e	307 ± 12 a	378 ± 13 a	375 ± 17 a	2.47 ± 0.50 ef	0.2633 ± 0.1102 d
DS12	63928 ± 5490 ab	786 ± 193 b	1125 ± 147 c	1321 ± 38 d	3.10 ± 0.72 cdef	0.2067 ± 0.1026 cd

Note: The different lowercase letters indicate that the difference was significant at the 0.05 level; n = 3.

## Data Availability

The sequence data from the aquatic fungi of in Yunnan Yunnan Dashanbao Black-necked Crane National Nature Reserve were deposited in the Sequence Read Archive of the NCBI under accession number PRJNA1205548.

## Supplementary Materials

The following supporting information can be downloaded at https://www.mdpi.com/article/10.3390/jof11070526/s1, Table S1: OTUs obtained from the 36 samples of aquatic fungi in Yunnan Dashanbao Black-necked Crane National Nature Reserve. Table S2: Abundance of aquatic fungi at 12 sites at phylum level. Table S3: Prediction of the nutritional and functional groups of fungal communities in different samples by FUNGuild. Table S4: Correlation Parameters of Fungal Community Structure with Environmental Factors. Figure S1: Location of 12 sampling sites in the Dashanbao Nature Reserve. Figure S2: Representative sampling sites. Figure S3: Hierarchical cluster dendrogram of 12 sites grouped by environmental variable similarities. Figure S4: Rarefaction curves of aquatic fungi of 36 samples at 12 sites based on Sobs index on OTU level. Figure S5: The aquatic fungal composition and distribution of 12 sites in Dashanbao Nature Reserve at genus level.
