# Supplementary material for: Relationship Between Aquatic Fungal Diversity in Surface Water and Environmental Factors in Yunnan Dashanbao Black-Necked Crane National Nature Reserve, China
Source: J Fungi (Basel). 2025 Jul 16;11(7):526. doi: 10.3390/jof11070526 (PMC12299766; doi:10.3390/jof11070526)
Supplement: Supplementary file 1 [file jof-11-00526-s001.zip › Figure S3 Hierarchical cluster dendrogram of 12 sites grouped by environmental variable similarities. .pdf]

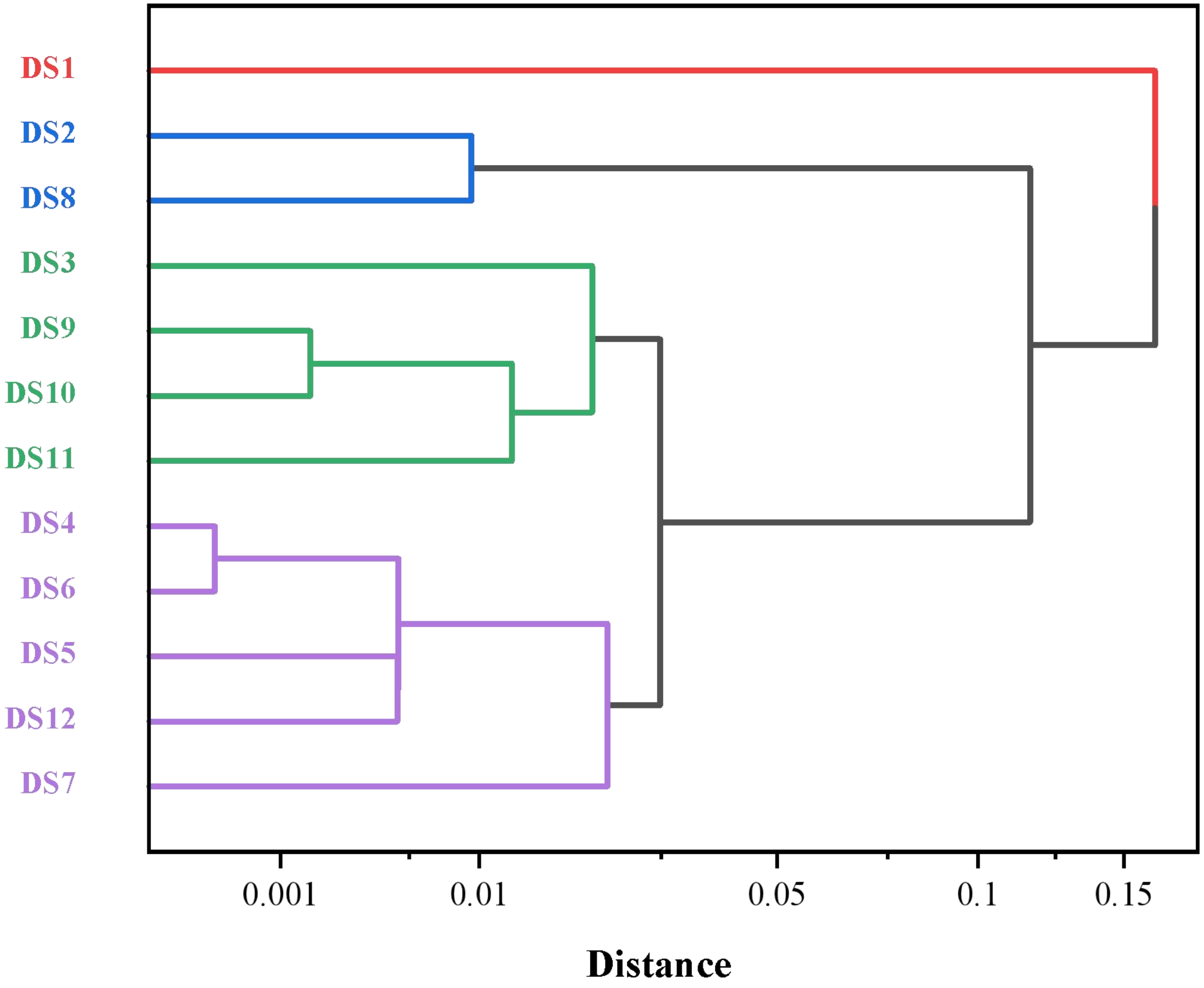

Figure S3: Hierarchical cluster dendrogram of 12 sites grouped by environmental variable similarities.
