# Supplementary material for: Relationship Between Aquatic Fungal Diversity in Surface Water and Environmental Factors in Yunnan Dashanbao Black-Necked Crane National Nature Reserve, China
Source: J Fungi (Basel). 2025 Jul 16;11(7):526. doi: 10.3390/jof11070526 (PMC12299766; doi:10.3390/jof11070526)
Supplement: Supplementary file 1 [file jof-11-00526-s001.zip › Figure S4 Rarefaction curves of aquatic fungi of 36 samples at 12 sites based on Sobs index on OTU level.pdf]

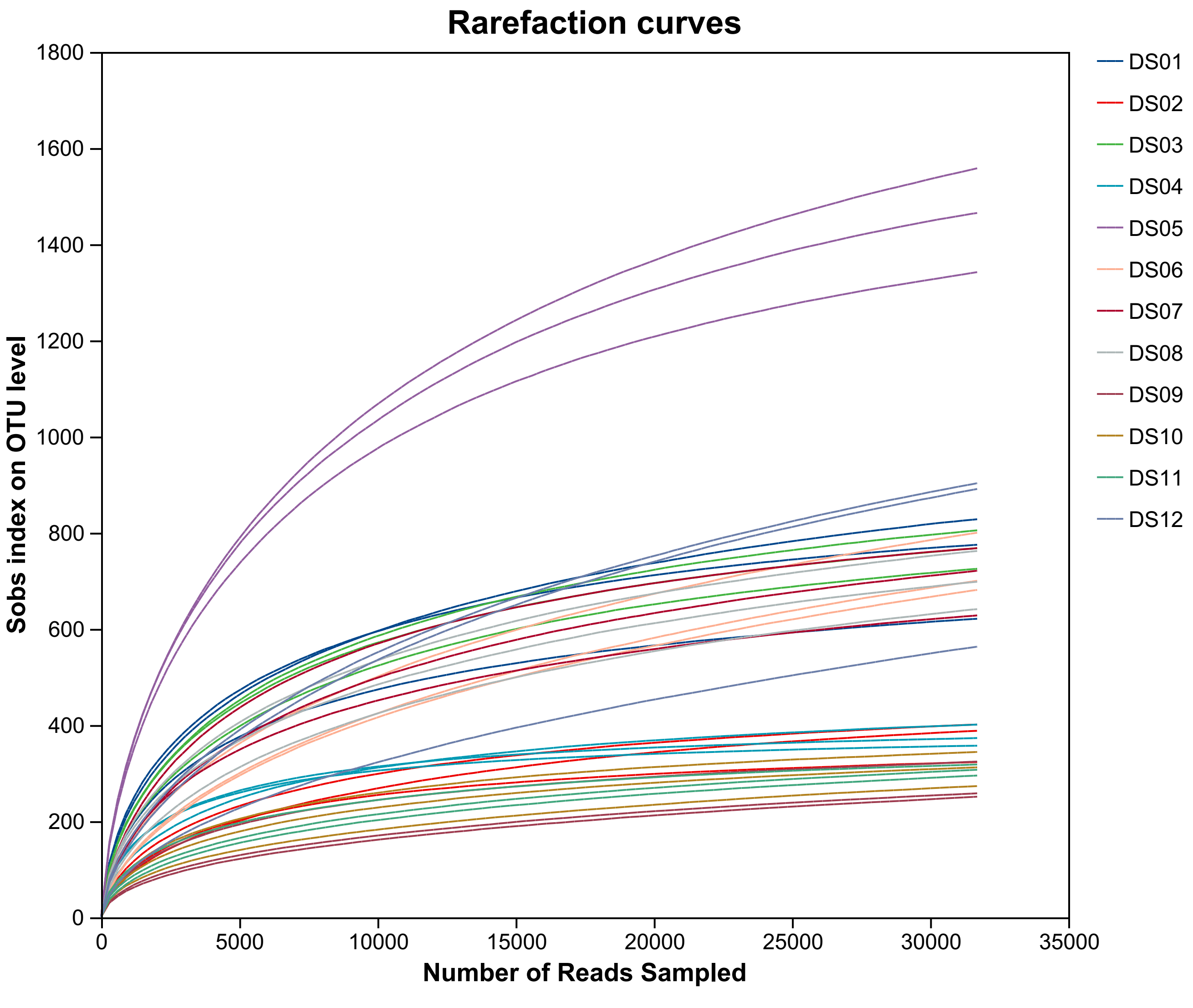

Figure S4 Rarefaction curves of aquatic fungi of 36 samples at 12 sites based on sobs index. The abscissa represents the number of reads, and the ordinate represents the Sobs index on OTU level
