# Supplementary material for: Relationship Between Aquatic Fungal Diversity in Surface Water and Environmental Factors in Yunnan Dashanbao Black-Necked Crane National Nature Reserve, China
Source: J Fungi (Basel). 2025 Jul 16;11(7):526. doi: 10.3390/jof11070526 (PMC12299766; doi:10.3390/jof11070526)
Supplement: Supplementary file 1 [file jof-11-00526-s001.zip › Table S4. Significant correlations between dominant fungal genera and environmental factors.pdf]

Table S4. Significant correlations between dominant fungal genera and environmental factors

Genus	TN (r)	DO (r)	pH (r)	TP (r)	Statistical significance
Mrakia	-0.75	-0.54	-0.83	-	p <0.05
Leucosporidium	-0.58	-0.68	-0.62	-	p <0.05
Filobasidium	-0.76	-0.55	-0.79	-	p <0.01
Cladosporium	-0.65	-0.60	-0.79	-	p <0.05
Monodictys	-0.70	-0.58	-0.75	-	p<0.05
Tausonia	-	-0.63	-0.72	-	p<0.05
Didymella	-0.59	-	-0.64	+0.71	p<0.001
Vishniacozyma	-0.72	-	-0.74	-	p<0.05
Cystofilobasidium	-0.69	-	-0.77	-	p<0.05
Holtermanniella	-0.66	-	-0.70	-	p<0.05
Udeniomyces	-0.61	-	-0.68	-	p<0.05
Aspergillus	-0.63	-	-0.71	-	p<0.05
