# Supplementary figures and images for: Relationship Between Aquatic Fungal Diversity in Surface Water and Environmental Factors in Yunnan Dashanbao Black-Necked Crane National Nature Reserve, China
Source: J Fungi (Basel). 2025 Jul 16;11(7):526. doi: 10.3390/jof11070526 (PMC12299766; doi:10.3390/jof11070526)

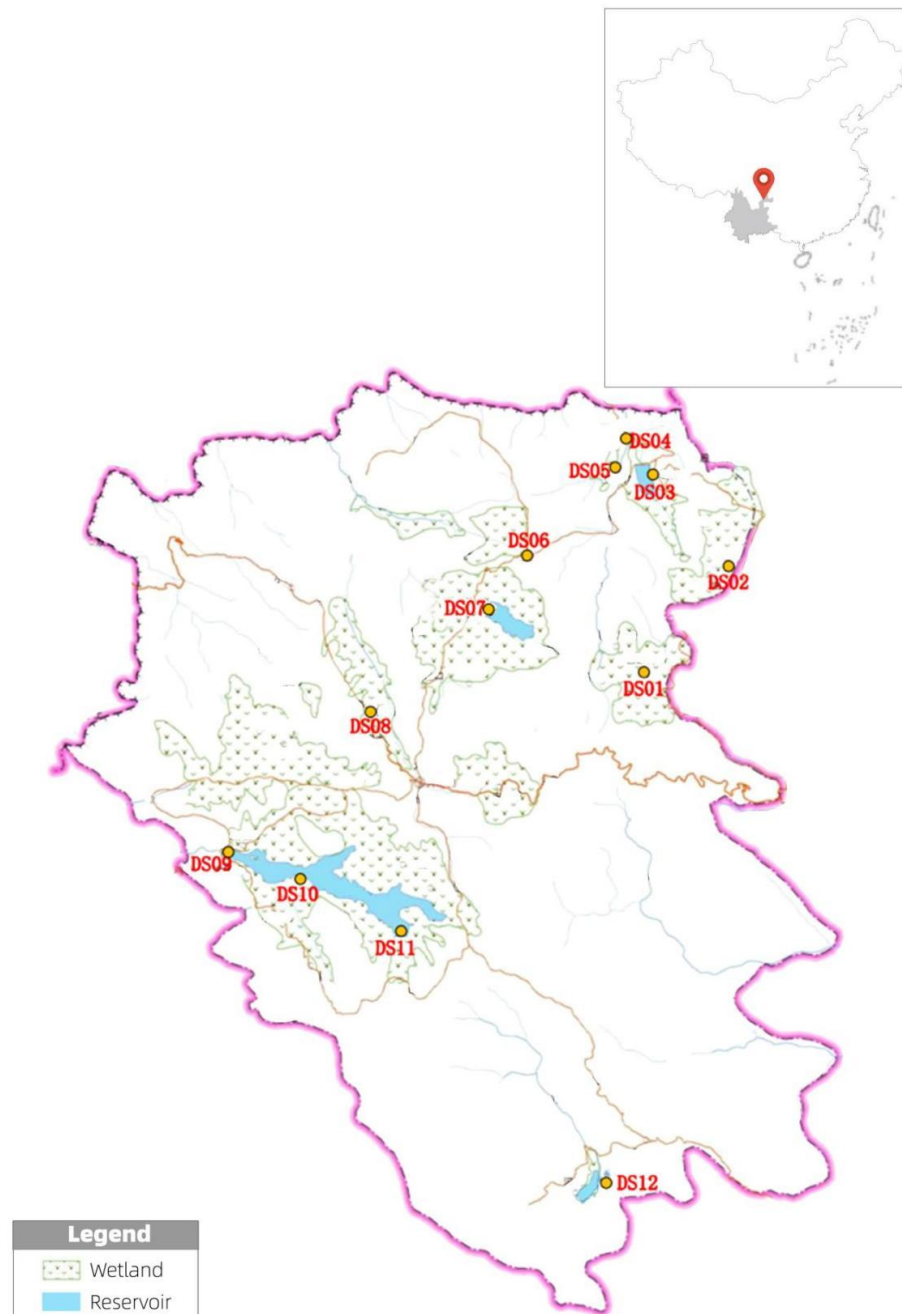

Figure S1. Location of 12 sampling sites in Dashanbao Nature Reserve.

Supplement: Supplementary file 1 [file jof-11-00526-s001.zip › Figure S1. Location of 12 sampling sites in the Dashanbao Nature Reserve..pdf]

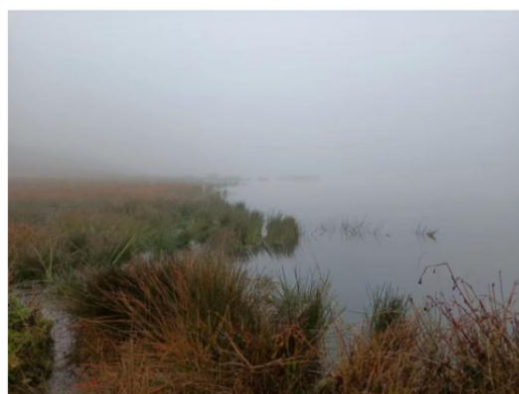

**DS01**

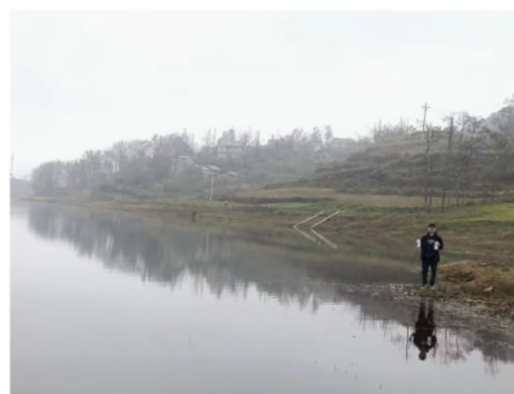

**DS03**

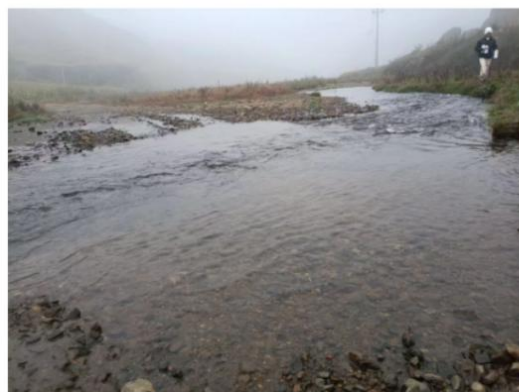

**DS05**

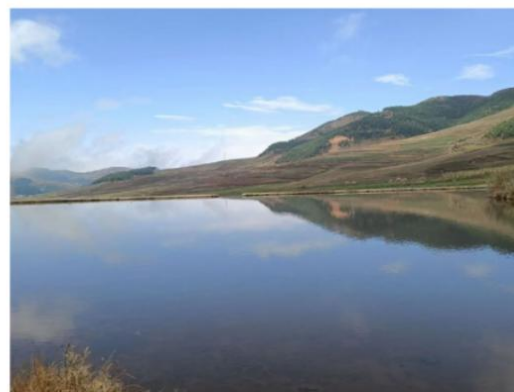

**DS06**

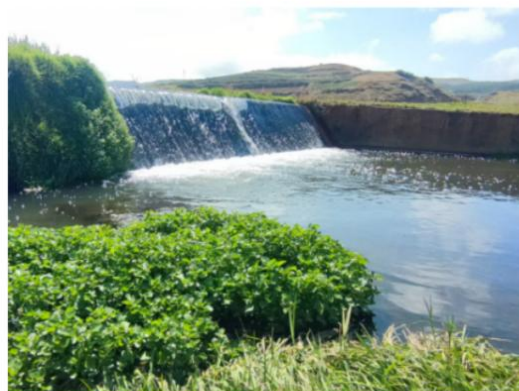

**DS08**

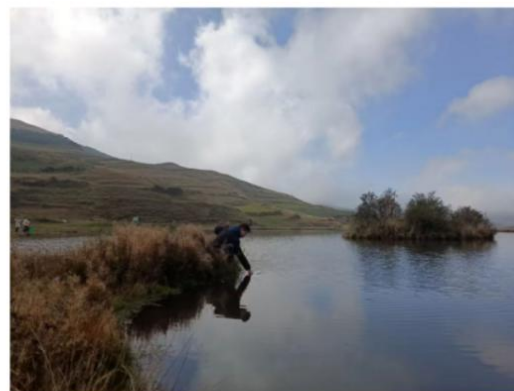

**DS10**

**Figure S2.** Representative sampling sites.

Supplement: Supplementary file 1 [file jof-11-00526-s001.zip › Figure S2. Representative sampling sites..pdf]
